# ERP evidence for the control of emotional memories during strategic retrieval

**DOI:** 10.3758/s13415-017-0509-9

**Published:** 2017-05-08

**Authors:** Jane E. Herron

**Affiliations:** Cardiff University Brain Research Imaging Centre (CUBRIC), School of Psychology, Cardiff University, Cardiff, CF24 4HQ Wales UK

**Keywords:** Cognitive control, Emotion, Episodic memory, ERP, Recollection

## Abstract

Neural evidence for the strategic retrieval of task-relevant ‘target’ memories at the expense of less relevant ‘nontarget’ memories has been demonstrated across a wide variety of studies. In ERP studies, this evidence consists of the ERP correlate of recollection (i.e. the ‘left parietal old/new effect’) being evident for targets and attenuated for nontargets. It is not yet known, however, whether this degree of strategic control can be extended to emotionally valenced words, or whether these items instead reactivate associated memories. The present study used a paradigm previously employed to demonstrate the strategic retrieval of neutral words (Herron & Rugg, *Psychonomic Bulletin and & Review, 10*(3), 703-–710, 2003b) to assess the effects of stimulus valence on behavioural and event-related potential (ERP) measures of strategic retrieval. While response accuracy and reaction times associated with targets were unaffected by valence, negative nontargets and new items were both associated with an elevated false alarm rate and longer RTs than their neutral equivalents. Both neutral and negative targets and nontargets elicited early old/new effects between 300 and 500 ms. Critically, whereas neutral and negative targets elicited robust and statistically equivalent left parietal old/new effects between 500 and 800 ms, these were absent for neutral and negative nontargets. A right frontal positivity associated with postretrieval monitoring was evident for neutral targets versus nontargets, for negative versus neutral nontargets, and for targets versus new items. It can therefore be concluded that the recollection of negatively valenced words is subject to strategic control during retrieval, and that postretrieval monitoring processes are influenced by emotional valence.

Episodic memory is memory for personally experienced events and is often accompanied by the recollection of contextual information such as time, place, and sensory details. It is widely accepted that control processes help us to navigate episodic memory, enabling us to constrain retrieval attempts in order to selectively recollect memories relevant to current goals. This concept has been expressed within cognitive theories of memory in various ways, such as ‘descriptors’ that guide a memory search (Burgess & Shallice, 1996), ‘context bias’ mechanisms that influence the processing of stimuli so as to facilitate retrieval from a particular context (Anderson & Bjork, 1994), cue-bias processes that optimise the cue-memory trace by specifying relevant contextual features (Mecklinger, 2010), and ‘retrieval orientations’ that guide retrieval searches towards specific contexts (Rugg & Wilding, 2000). Supporting these theories, a significant number of event-related potential (ERP) recognition memory studies have now provided evidence that established neural correlates of recollection are evident for ‘target’ test items that were studied in an experimenter-designated encoding context, whereas these are attenuated or even eliminated for ‘nontarget’ items studied in an alternative context (Dywan, Segalowitz, & Arsenault, 2002; Dywan, Segalowitz, & Webster, 1998; Dzulkiflil, Herron, & Wilding, 2006; Evans, Wilding, Hibbs, & Herron, 2010; Herron & Rugg, 2003a, 2003b; Rosburg, Johansson, & Mecklinger, 2013; Rosburg, Mecklinger, & Johansson, 2011; Wilding, Fraser, & Herron, 2005). All of these studies have employed stimuli that are relatively neutral with respect to emotional valence. The principal aim of this study is to examine whether emotionally arousing negative stimuli are also subject to these strategic retrieval control processes, and more specifically, whether recollection of these items can be similarly attenuated when designated as nontargets.

The influence of emotion on episodic memory is complex and, in some sense, contradictory. Unpleasant memories can spontaneously intrude upon our thoughts, despite the uncomfortable feelings that they arouse, and this feeling of discomfort compels us to try to suppress these memories from consciousness. These characteristics make negatively valenced items intriguing stimuli to examine from the perspective of memory control. Behavioural studies have shown that recognition memory is sometimes superior for emotionally valenced than for neutral stimuli (Adelman & Estes, 2013; Inaba, Nomura, & Ohira, 2005; Kensinger & Corkin, 2003, 2004; Ochsner, 2000), although this recognition enhancement has not consistently been found (Doerksen & Shimamura, 2001; Leiphart, Rosenfeld, & Gabrieli, 1993; Maratos, Allan, & Rugg, 2000) and valence has sometimes been confounded with arousal (comment by Mather & Sutherland, 2009, but see Adelman & Estes, 2013). Emotionally valenced items tend to be associated with a more liberal response bias than neutral items, with both studied and unstudied emotional items attracting more recognition responses (Dougal & Rotello, 2007; Thapar & Rouder, 2009; Windmann & Kutas, 2001). It has been suggested that this may be due to greater semantic cohesiveness amongst emotionally valenced stimuli (Maratos et al., 2000; White, Kapucu, Bruno, Rotello, & Ratcliff, 2014). Furthermore, while some researchers have reported enhanced source memory for emotionally valenced items (D’Argembeau & Van der Linden, 2004; Doerksen & Shimamura, 2001; Kensinger & Corkin, 2003), these items were associated with deficits in source monitoring processes across a series of four experiments by another research team (Cook, Hicks, & Marsh, 2007). There is some evidence to suggest that emotional valence enhances source retrieval when retrieving contextual information intrinsic to the stimuli such as colour or location (D’Argembeau & Van der Linden, 2004; Doerksen & Shimamura, 2001; Kensinger & Corkin, 2003), but not when retrieving extrinsic information that is not part of the stimuli such as encoding task (Kensinger & Schacter, 2006; Mather, 2007).

A number of studies have employed ERPs to examine the effects of word valence on neural correlates of episodic memory (Inaba et al., 2005; Maratos et al., 2000; Windmann & Kutas, 2001). Maratos et al. (2000) reported that two established ERP correlates of recognition memory (see Friedman & Johnson, 2000; Rugg & Curran, 2007, for reviews) were qualitatively the same for negative and for neutral words; an early frontal effect (300–500 ms) associated with familiarity-based recognition was unaffected by valence, while an effect at left parietal sites (500–800 ms) associated with recollection was smaller in size for negative than for neutral items. This was because ERPs associated with unstudied items were more positive going for negative than for neutral items while amplitudes of ERPs elicited by recognised items were equivalent across valence. A late right frontal old/new effect (800–1,400 ms) that has been linked to episodic source monitoring (Cruse & Wilding, 2009; Donaldson & Rugg, 1998; Johnson, Kounios, & Nolde, 1997; Wilding & Rugg, 1996) was evident for neutral and not for negative items (Maratos et al., 2000). Similarly, Windmann and Kutas (2001) found no effect of valence on ERP old/new effects between 300–500 ms and 500–700 ms. Conversely, Inaba et al. (2005) examined ERPs associated with the recognition of negative, positive, and neutral words and found that while valence did not influence ERPs associated with unstudied items, the positivity elicited by recognised items between 400 and 700 ms at left parietal sites was greatest for negative items and smallest for neutral items. The authors proposed that the superior recognition memory observed for negative words could be attributed to this ERP enhancement for recognised items.

Further ERP studies have examined the influence of emotional valence on memory effects for neutral stimuli embedded in neutral or valenced contexts at encoding. Maratos and Rugg (2001) reported that left parietal and right frontal old/new effects were larger for words previously embedded in negative sentences during recognition but not during a source memory test, while Ventura-Bort et al. (2016) reported larger early frontal old/new effects for objects encoded in a negative context than those encoded in a pleasant or a neutral context and a parietal old/new effect between 400 and 700 ms for objects encoded in a valenced context (both negative and positive) but not in a neutral context. A. P. Smith, Dolan, and Rugg (2004) examined old/new effects for objects which were associated with neutral or valenced backgrounds at study and found that while valence did not influence the left parietal or right frontal effects, additional effects for objects associated with valenced backgrounds were evident. These included a 300–500 ms lateral posterior effect and an 800–1,900 ms left temporo-central effect. Jaeger and colleagues have also found effects of emotional valence on ERP correlates of incidental (Jaeger, Johnson, Corona, & Rugg, 2009) and implicit (Jaeger & Rugg, 2012) memory for pictures.

Turning to memory control, the left parietal old/new ERP effect has been employed as a marker of recollection in memory paradigms in which it is strategically beneficial to prioritise the retrieval of target memories at the expense of nontarget memories. These paradigms borrow from the ‘exclusion’ task introduced by Jacoby (1991), in which items are encoded in two different contexts and then presented together with unstudied items at test. A positive recognition response is required only for test items from a single ‘target’ context while items from the alternate ‘nontarget’ context are responded to on the same key as unstudied items. Although both target and nontarget responses can theoretically be made on the basis of recollection—‘recall-to-reject’ in the case of nontargets (Clark, 1992)—a number of ERP studies have reported that when retrieval accuracy associated with targets is high, the left parietal old/new effect elicited by nontargets is attenuated or even eliminated (Dywan et al., 2002; Dywan et al., 1998; Dzulkiflil et al., 2006; Evans et al., 2010; Herron & Rugg, 2003a, 2003b; Wilding et al., 2005). On the basis of these findings, it has been proposed that when memory for targets is high and reliable, participants adopt a strategy in which retrieval efforts are focused on items from the target context, which results in reduced recollection of items from the nontarget context (Herron & Rugg, 2003b).

Although this proposal accounts for the pattern of target and nontarget left parietal old/new effects across a number of studies, Rosburg and colleagues (Rosburg et al., 2013; Rosburg et al., 2011) have proposed that the ‘ease of nontarget accessibility’ also determines whether nontargets will elicit the ERP correlate of recollection. According to this argument, bottom-up mechanisms can operate independently of top-down strategic control, with memories being automatically reactivated for certain kinds of nontarget. Although both accounts acknowledge the importance of proactive ‘top-down’ strategic retrieval processes that prioritise target recollection, and accept that this often directly results in an attenuation of nontarget recollection, it remains unknown whether the suppression of nontarget recollection during strategic retrieval extends to emotionally valenced items. It is conceivable that, even under retrieval conditions that promote the strategic retrieval of target information, emotionally valenced cues associated with higher arousal values may elicit the bottom-up reactivation of nontarget memories as described by Rosburg et al. (2013; Rosburg et al., 2011). Examining ERP correlates of recollection for negative nontargets in the present study will allow this issue to be addressed.

Due to the controversies surrounding ‘reverse inference’ (i.e. assuming the degree to which a cognitive process is invoked via the presence/absence of its neural correlate), it is worth briefly revisiting the strength of association between recollection and the left parietal old/new effect (for reviews, see Friedman & Johnson, 2000; Mecklinger, 2000; Rugg & Curran, 2007; Rugg & Yonelinas, 2003). This effect is characterised by greater positivity elicited by correctly identified studied than unstudied test items which is maximal at left parietal scalp sites between 500 and 800 ms after test item presentation. The amplitude of the effect is larger for recognised items accompanied by retrieval of source information (Wilding, Doyle, & Rugg, 1995; Wilding & Rugg, 1996), correlates with the number and accuracy of source judgments made (Vilberg, Moosavi, & Rugg, 2006; Wilding, 2000), is larger when participants subjectively report that they can ‘remember’ an item as opposed to having an acontextual sense of familiarity (Duarte, Ranganath, Winward, Hayward, & Knight, 2004; Duzel, Yonelinas, Mangun, Heinze, & Tulving, 1997; M. E. Smith, 1993; Vilberg et al., 2006; Vilberg & Rugg, 2009), and is reduced in amplitude both for patients with impaired recollection and in pharmacological studies in which recollection is impaired (Curran, DeBuse, Woroch, & Hirshman, 2006; Duzel, Vargha-Khadem, Heinze, & Mishkin, 2001; Mecklinger, von Cramon, & Matthes-von Cramon, 1998; Potter, Pickles, Roberts, & Rugg, 1992; Rugg, Roberts, Potter, Pickles, & Nagy, 1991; M. E. Smith & Halgren, 1989; Tendolkar et al., 1999; Vargha-Khadem et al., 1997). The strength of association between the left parietal old/new effect and recollection (and the broad consensus regarding this) is such that the effect has been frequently used as a proxy for recollection (Bergstrom, de Fockert, & Richardson- Klavehn, 2009a, 2009b; Bergstrom, Velmans, de Fockert, & Richardson-Klavehn, 2007; Czernochowski, Mecklinger, Johansson, & Brinkmann, 2005; Depue et al., 2013; Dzulkiflil et al., 2006; Dzulkifli & Wilding, 2005; Evans et al., 2010; Hanslmayr, Leipold, Pastotter, & Bauml, 2009; Herron & Rugg, 2003a, 2003b; Herron & Wilding, 2005; Mecklinger, Parra, & Waldhauser, 2009). The present study also takes this approach, the primary aim being to discover whether the recollection of emotionally valenced items can be controlled. The key question is whether neural activity elicited by emotionally negative nontargets would reveal the same degree of attenuation of the left parietal old/new effect as that observed for neutral nontargets.

A second ERP modulation relevant to this study is the ‘right frontal old/new effect’, which takes the form of enhanced positivity elicited by studied items at right frontal sites after 800 ms, and which has been linked to postretrieval monitoring processes (Cruse & Wilding, 2009; Donaldson & Rugg, 1998; Johnson et al., 1997; Wilding & Rugg, 1996). It has been characterised as a correlate of episodic source monitoring because it is larger when source judgments are required when compared with item recognition (Johansson, Stenberg, Lindberg, & Rosen, 2002; Johnson et al., 1997; Senkfor & Van Petten, 1998; Van Petten, Senkfor, & Newberg, 2000) and is also larger for correct than for incorrect source judgments (Wilding & Rugg, 1996). However, the case for reverse inference is not as strong. This is both because the effect has not always been shown to predict source accuracy (Senkfor & Van Petten, 1998; Van Petten et al., 2000) and because it has also been observed in tasks that do not require episodic retrieval (Hayama, Johnson, & Rugg, 2008; Hayama & Rugg, 2009). It has therefore been proposed that this effect may either reflect more generic monitoring/decisional processes irrespective of the memory system involved (Hayama & Rugg, 2009) or that it may encompass different postretrieval processing operations reflecting the functional heterogeniety of right prefrontal cortex (Cruse & Wilding, 2009; Wilding & Ranganath, 2011). It will be of interest to observe the impact of emotional valence on the right-frontal effect in the present study as behavioral data regarding the influence of valence on source monitoring has been mixed.

To summarise, the principal aim of the study is to examine whether established ERP correlates of strategic retrieval extend to negatively valenced stimuli. The key hypotheses are as follows: (i) if the recollection of negative items is subject to the same degree of strategic control as that previously observed for neutral items, then the left parietal old/new effect will show equivalent levels of attenuation for both classes of nontargets with respect to targets; (ii) if source monitoring is impaired for negative items, then ERP correlates of postretrieval monitoring will be smaller for these items at right frontal sites post-800 ms; (iii) if the recollection of extrinsic contextual information is unaffected by valence, then no effects of valence will be observed for behavioral and ERP measures of target recollection. The findings will inform models of strategic retrieval by providing novel information regarding the kind of memory contents that are subject to retrieval control. They will also inform models of emotional memory by providing new behavioral and neural evidence regarding the impact of stimulus valence on the retrieval of extrinsic contextual information for both goal-relevant and nontarget stimuli.

## Method

Participants were drawn from the undergraduate population studying psychology at Cardiff University and participated on a voluntary basis in return for course credit after giving informed consent. Eighteen right-handed native English speakers ages 18 to 28 years (mean age: 20 years, 12 women) participated in the experiment and all participants contributed data to the analyses reported below. The sample size was based upon that used by Herron and Rugg (2003b; *N* = 16 in each of two experiments) who employed the same experimental design. Ethical approval was for the study granted by Cardiff University’s School of Psychology ethics committee.

Stimuli consisted of 240 words selected from the ANEW database (Bradley & Lang, 1999). All words were between three and 10 letters in length and had a word frequency of between one and 50 occurrences per million (Kucera & Francis, 1967). Average word length and frequency were 6.58 and 13.14 for negative words, and 5.84 and 14.61 for neutral words, respectively. Half of the words had a negative emotional valence rating of between 1 and 3, and half had a neutral valence rating of between 4.5 and 6.5 (according to the ANEW database). The mean valence rating for negative words was 2.25 (*SD* = 0.46), whereas the mean valence rating for neutral words was 5.49 (*SD* = 0.52), a difference shown to be significant *t* = 53.58, *p* < .001, Cohen’s *d*_*z*_ = 6.92. The mean arousal rating for negative words was 5.86 (*SD* = 0.94), whereas the mean arousal rating for neutral words was 4.18 (*SD* = 0.70), a difference again shown to be significant *t* = 15.73, *p* < .001, Cohen’s *d*_*z*_ = 2.03. All stimuli were presented in white letters on a black background, on a monitor located 1.2 m from the participant. All stimuli were presented at central fixation and subtended maximum visual angles of 0.5° (vertical) and 2.2° (horizontal). The experiment consisted of Study 1, Study 2, and an exclusion memory test. Forty neutral and 40 negative words were randomly intermixed in Study 1. Participants were required to verbally generate a meaningful sentence incorporating each word before proceeding to the next item. A further 40 neutral and 40 negative words were randomly intermixed and presented in Study 2. Participants made ‘very pleasant’/’fairly pleasant’/’fairly unpleasant’/’very unpleasant’ judgments to each item by button press.^1^ At test, all items from Study 1 and Study 2 were randomly intermixed with 40 neutral and 40 negative new words. Participants were instructed to respond on one response key to items from Study 2 only (‘targets’) and to respond on an alternate key both to new items and to items from Study 1 (‘nontargets’).

In Study 1, each trial began with an asterisk which was visible in the centre of the screen for 100 ms and followed by a blank screen for 122 ms. Study words were then presented for 300 ms, after which the monitor was blanked while participants spoke the sentence aloud then pressed a response key to initiate the next trial. The trial sequence was the same for items in Study 2, with the exception that participants made their pleasantness judgments by means of a four-way button press which initiated the next trial. Very pleasant/fairly pleasant judgments were made with two fingers of the same hand, whereas fairly unpleasant/very unpleasant judgments were made with two fingers of the alternate hand. Each test trial began with an asterisk presented at central fixation for 100 ms, after which the screen was blanked for 122 ms. The test word was then presented for 300 ms after which the screen was blanked while participants responded on one key to items from Study 2 (i.e. those items that had been rated for pleasantness) or on a second key both to new items and to items from Study 1 (i.e. those that had been encoded in the sentence generation task). The response to each item initiated the next trial after an interval of 1,500 ms during which the screen remained blank. Two-minute intervals separated Study 1 and Study 2, and Study 2 and test, during which instructions were given to the participant for the upcoming task. Items were rotated across participants so that they served as Study 1 items, Study 2 items, and unstudied items an equal number of times. The hands used for target and nontarget/new responses were also counterbalanced across participants.

### EEG acquisition

EEG was recorded using a Biosemi active electrode system from 32 recording locations based on the International 10-20 system (Jasper, 1958) including midline (Fz, Cz, Pz, Oz) and left/right hemisphere locations (FP1/FP2, F7/F8, F5/F6, F3/F4, F1/F2, T7/T8, C5/C6, C3/C4, C1/C2, P7/P8, P5/P6, P3/P4, P1/P2, O1/O2). Additional electrodes were placed on the mastoid processes. Electrooculogram (EOG) was recorded from above and below the left eye (VEOG) and from the outer canthi (HEOG). Electroencephalogram (EEG; range DC-419 Hz; sampling rate 2048 Hz) was acquired referenced to linked electrodes located midway between POz and PO3/PO4, respectively, and was rereferenced off-line to linked mastoids. Data were high-pass filtered off-line (0.03 Hz) and down-sampled to 170 Hz, resulting in a total epoch length of 1,505 ms with a 102 ms baseline relative to which all mean amplitudes were computed. Trials containing large EOG artefact were rejected, as were trials containing A/D saturation or baseline drift exceeding ±80 mV. Other EOG blink artefacts were corrected using a linear regression estimate (Semlitsch, Anderer, Schuster, & Presslich, 1986). A 7-point binomially weighted smoothing filter was applied prior to analysis.

## Results

All analyses included the Greenhouse–Geisser correction for nonsphericity where necessary (Greenhouse & Geisser, 1959). The term ‘correct rejections’ (or ‘CRs’) refers to unstudied items correctly classified at test. The term ‘target hits’ refers to Study 2 items correctly classified at test, and ‘nontarget CRs’ to Study 1 items correctly classified at test.

### Behaviour

Analysis of self-report pleasantness judgments during Study 2 showed that an average of 97% (*SD* = 5) of negative stimuli attracted ‘fairly unpleasant’ or ‘very unpleasant’ responses, while 86% (*SD* = 11) of neutral items attracted ‘fairly pleasant’ or ‘very pleasant’ responses. It should be noted here that there was no neutral response option. The four response options were scored (*very unpleasant* =1, *fairly unpleasant* = 2, *fairly pleasant* = 3, *very pleasant* = 4) and analysed for each stimulus type. Negative items received a mean valence score of 1.52 (*SD* = 0.26) whereas neutral items received a mean valence score of 3.04 (*SD* = 0.17). A paired *t* test revealed this difference to be significant *t*(1, 17) = 17.49, *p* < .001, Cohen’s *d*_*z*_ = 4.12.

Table 1 shows the response accuracy and associated RTs for studied and new items separated by emotional valence (negative and neutral). The likelihood of a correct response to all items (both negative and neutral) was greater than the likelihood of an incorrect response (*t*s > 7, *p*s < .001, in each case).

**Table 1:** Response accuracy and associated RTs for target, nontarget and new items separated by emotional valence (*SD*s in brackets)

	Accuracy	Reaction Time
Negative		
Targets	.81 (.13)	1509 (426)
Nontargets	.73 (.14)	1733 (518)
New	.87 (.12)	1352 (430)
Neutral		
Targets	.83 (.11)	1413 (415)
Nontargets	.85 (.12)	1420 (285)
New	.97 (.03)	1216 (355)

Discrimination (P_r_) between targets and new items (target hits – new item false alarms) was .80 and .68 for neutral and negative items, respectively, and measures of response bias (B_r_ = new item false alarms/(1 - P_r_)) between targets and new items were .15 and .41 for neutral and negative items respectively (Feenan & Snodgrass, 1990). ANOVA of the accuracy data employed the design Response Type (target hits/nontarget CRs/new CRs) × Valence (neutral/negative) and gave rise to a main effect of Response Type, *F*(1.9, 32.6) = 12.68, *p* < .001, η_p_^2^ = 0.43, a main effect of Valence, *F*(1, 17) = 28.80, *p* < 0.001, η^2^_p_ = 0.63, and a Response Type x Valence interaction *F*(1.9, 32.5) = 5.03, *p* < .05, η_p_^2^ = 0.23. Subsidiary pairwise comparisons (Bonferroni-corrected *p* values = .017) revealed that the hit rate was statistically equivalent for negative and neutral targets, whereas the correct rejection rate was higher for neutral words than for their negative equivalents for both nontargets, *F*(1, 17) = 20.98, *p* < .001, η_p_^2^ = 0.55, and for new items, *F*(1, 17) = 13.79, *p* = .002, η_p_^2^ = 0.45. The parallel ANOVA of RTs associated with correct responses gave a main effect of Response Type, *F*(1.9, 31.6) = 31.88, *p* < .001, η_p_^2^ = 0.65, a main effect of Valence, *F*(1, 17) = 19.62, *p* < .001, η_p_^2^ = 0.54, and a Response Type × Valence interaction, *F*(1.9, 32.1) = 5.47, *p* < .05, η_p_^2^ = 0.24. Subsidiary pairwise comparisons (Bonferroni-corrected *p* values = .017) revealed statistically equivalent RTs for negative and neutral target hits, whereas RTs were faster for neutral than for negative items for both nontarget CRs, *F*(1, 17) = 17.39, *p* = .001, η_p_^2^ = 0.51, and new item CRs, *F*(1, 17) = 7.28, *p* = .015, η_p_^2^ = 0.30.

### ERPs

**Fig. 1:**
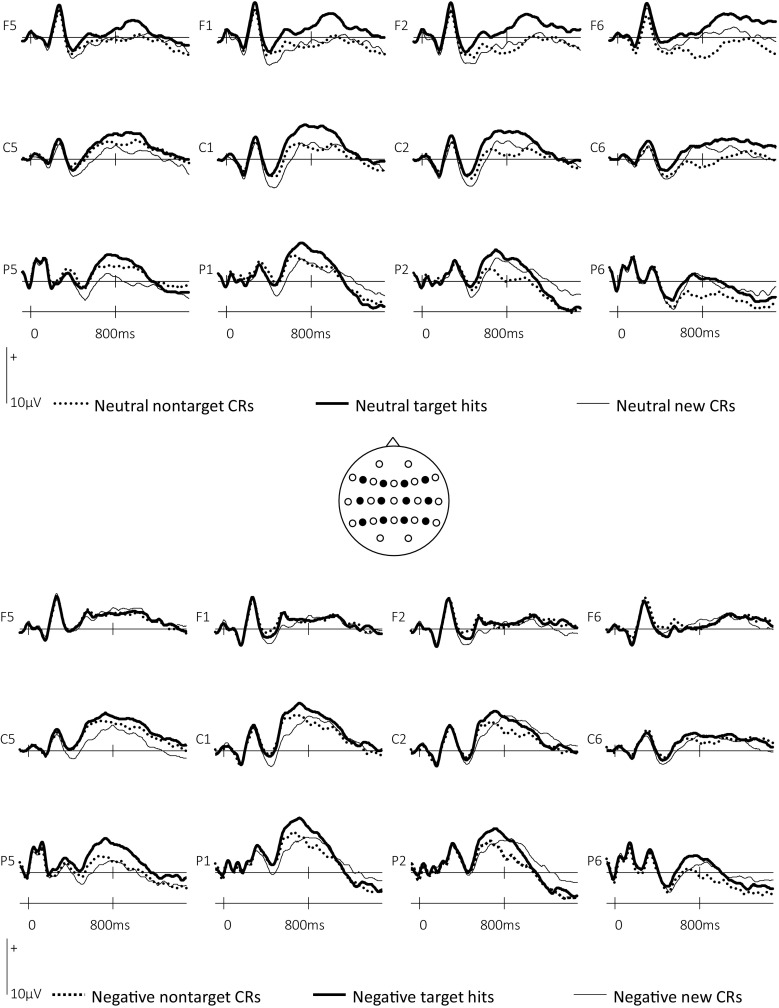
ERPs elicited by neutral and negative target hits, nontarget CRs, and new CRs at left frontal (F5, F1), right frontal (F6, F2), left central (C5, C1), right central (C6, C2), left posterior (P5, P1), and right posterior (P6, P2) electrode sites

ERPs associated with target hits, nontarget CRs and CRs and separated according to valence are shown in Fig. 1. The mean numbers of trials (minimum and maximum in parentheses) contributing ERPs for each response type were as follows: negative target hits: 30 (22–36), neutral target hits: 30 (19–36), negative nontarget CRs: 27 (18–35), neutral nontarget CRs: 31 (20–38), negative CRs: 32 (21–40), neutral CRs: 36 (31–39). Two sets of ERP analyses were performed. A set of global analyses incorporated data from a grid of 24 electrode sites distributed across the scalp (F7/F8, F5/F6, F3/F4, F1/F2, T7/T8, C5/C6, C3/C4, C1/C2, P7/P8, P5/P6, P3/P4, P1/P2) and included the factors of Response Type (target/nontarget/new), Valence, Anterior/Posterior, Hemisphere, and Site (inferior/midlateral/superior/midline). These were conducted on data from three epochs; 300–500 ms, 500–800 ms, and 800–1,400 ms. These epochs have been widely demonstrated in the ERP memory literature to capture three temporally and spatially dissociable episodic memory effects (Wilding & Ranganath, 2011); a 300–500-ms old/new effect with a midfrontal maximum related to familiarity-based recognition (Curran, 2000; Rugg et al., 1998; Woodruff, Hayama, & Rugg, 2006, but see Voss & Paller, 2006), a 500–800-ms old/new effect with a left parietal maximum considered to act as a reliable index of recollection (see above), and a right frontal effect between 800 and 1,400 ms thought to reflect postretrieval monitoring of either an episodic (Wilding & Rugg, 1996) or more generic (Hayama et al., 2008) nature. Finally, a fourth ERP old/new effect frequently evident in source memory paradigms and captured by the 800–1,400-ms epoch is the ‘late posterior negativity’ (LPN), a sustained negativity evident at parietal sites for studied items. This effect has been shown to incorporate both action monitoring and reconstructive episodic memory processes (Herron, 2007; Johansson & Mecklinger, 2003), and has most recently been characterised as a correlate of reconstructive source memory processes involved in both episodic and semantic memory (Mecklinger, Rosburg, & Johansson, 2016).

**Fig. 2:**
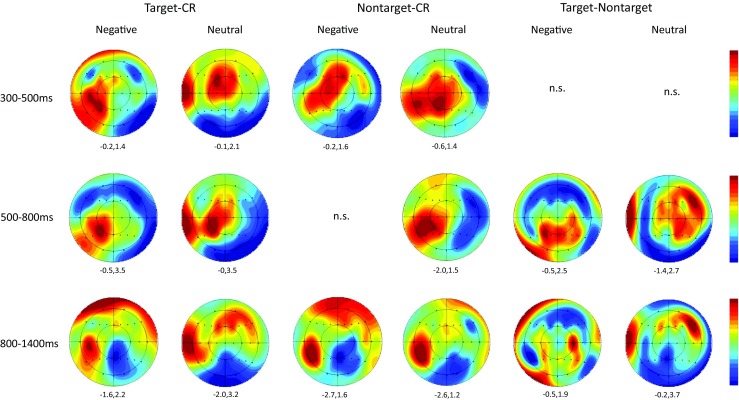
Voltage maps showing the scalp distributions of significant effects of Response Type obtained in each pairwise comparison in the 300–500-ms, 500–800-ms, and 800–1,400-ms epochs. Separate *scalp maps* are shown for *negative* and for *neutral* items. The *voltage bar* beside each map shows the correspondence between colour and maxima/minima, and max/min values specific to each contrast are displayed beneath each map. (Colour figure online)

**Fig. 3:**
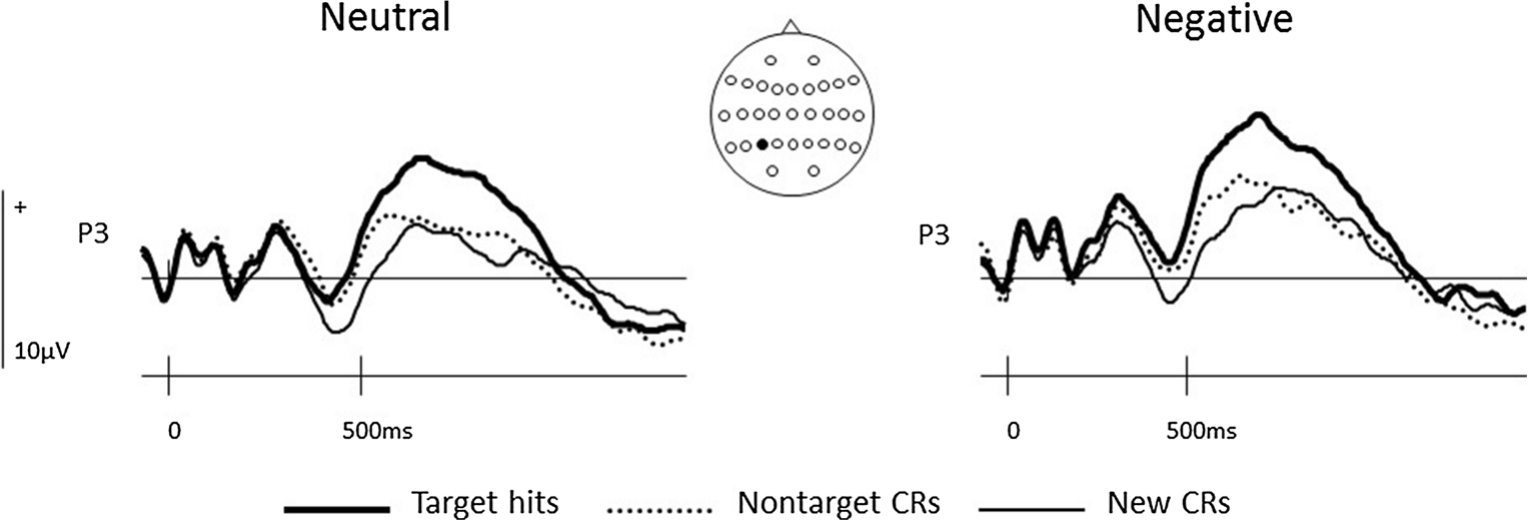
ERPs elicited by *neutral* and *negative target hits*, *nontarget CRs*, and *new CRs* at the left parietal scalp site (P3) closest to the maxima of the associated target hit–new CR effects as shown in Fig. 1

**Fig. 4:**
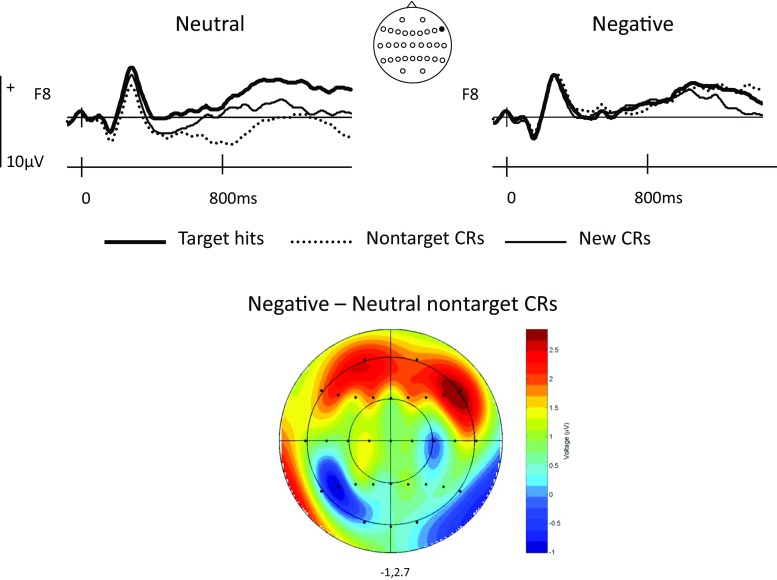
ERPs elicited by *neutral* and *negative target hits*, *nontarget CRs*, and *new CRs* at the right frontal scalp site (F8). The *scalp map* show the distribution of the *negative*–*neutral nontarget CR* effect between 800 and 1,400 ms. The *voltage bar* shows the correspondence between colour and effect magnitude (μv), and max/min is displayed beneath the map. (Colour figure online)

An additional set of planned ERP analyses motivated by the principal experimental hypotheses focused upon the modulation of left parietal and right frontal old/new effects. The former included data from the four left parietal scalp sites between 500 and 800 ms in order to assess the effects of Response Type and Valence on the left parietal old/new effect. These sites were selected both because many ERP studies have reported correlates of recollection maximal at left parietal scalp sites (e.g. Rugg & Allan, 2000; Rugg et al., 1998; Wilding & Sharpe, 2003) and because previous ERP studies of strategic recollection have performed targeted analyses at left parietal sites contained within this array (Dzulkifli & Wilding, 2006; Evans et al. 2010; Herron & Rugg, 2003b; Herron & Wilding, 2005; Wilding et al. 2005). Figure 2 confirms that the target hit/CR effects for both neutral and negative items showed a left parietal scalp distribution within this time window. Figure 3 indicates that neutral and negative target hits were more positive going than both CRs and nontarget CRs at left parietal sites, with little differentiation visually evident between the latter two response types. The second planned analysis focused on ERP data from right frontal sites between 800 and 1,400 ms to assess the impact of Response Type and Valence on the ERP correlate of postretrieval monitoring. These sites were selected on the basis of studies localising ERP correlates of postretrieval monitoring to right frontal sites (Cruse & Wilding, 2009; Donaldson & Rugg, 1998; Johnson et al., 1997; Wilding & Rugg, 1996) and more recent studies performing targeted analyses of these effects at the same (or subset of) right frontal sites as those analysed here (Beato, Boldini, & Cadavid, 2012; Boldini, Beato & Cadavid, 2013; Cadavid & Beato, 2016; Rosburg et al., 2011). Figure 4 indicates that neutral target hits were more positive going than either neutral CRs or neutral nontarget CRs at right frontal sites between 800 and 1,400ms. The three negative response types showed a smaller degree of differentiation, although negative targets and nontarget CRs were both more positive going than new CRs during this time window.

For all analyses, only main effects and highest order interactions involving the factor of Response Type are reported in the text (lower order effects of Response Type and effects of Valence in the absence of Response Type are reported in Table 2). Significant effects of Response Type were followed up with pairwise comparisons between targets and new CRs, nontargets and new CRs, and targets and nontargets which included the factors of Response Type, Valence, Anterior/Posterior, Hemisphere, and Site. Statistical post hoc tests were performed at the electrode site maxima indicated by highest order interactions in these pairwise comparisons to confirm whether or not the effects were statistically reliable.

**Table 2:** All significant effects of Response Type (RT) and Valence (VA) in the global ERP analyses incorporating factors of Hemisphere (HM), Anterior/Posterior (AP), and Site (ST)

	300–500	500–800	800–1400
Global:			
RT	*F*_(_1.6, 26.8) = 5.49*, η_p_^2^ = 0.24	*F*(1.4, 24.6) = 10.26**, η_p_^2^ = 0.38	*F*(1.6, 27.2) = 4.11*, η_p_^2^ = 0.19
VA	*F*(1, 17) = 18.45***, η_p_^2^ = 0.52	*F*(1, 17) = 25.48***, η_p_^2^ = 0.60	
RT × HM		*F*(1.3, 21.5) = 7.61**, η_p_^2^ = 0.31	*F*(2.0, 33.9) = 4.02*, η_p_^2^ = 0.19
RT × ST		*F*(2.5, 41.8) = 3.95*, η_p_^2^ = 0.19	
VA × AP	*F*(1.4, 23.0) = 7.93**, η_p_^2^ = 0.31		
VA × ST	*F*(1.1, 19.2) = 5.93*, η_p_^2^ = 0.26	*F*(1.1, 19.2) = 8.93**, η_p_^2^ = 0.34	
RT × AP × HM		*F*(2.6, 44.0) = 4.51*, η_p_^2^ = 0.21	*F*(3.0, 51.0) = 4.17**, η_p_^2^ = 0.20
RT × AP × ST		*F*(6.1, 104.5) = 2.49*, η_p_^2^ = 0.13	*F*(5.4, 91.2) = 3.48**, η_p_^2^ = 0.17
VA × AP × HM		*F*(1.9, 33.0) = 4.30*, η_p_^2^ = 0.20	
VA × AP × ST	*F*(3.1, 53.2) = 5.04**, η_p_^2^ = 0.23	*F*(4.1, 70.5) = 6.92***, η_p_^2^ = 0.29	*F*(4.2, 71.9) = 3.90**, η_p_^2^ = 0.19
VA × AP × HM × ST	*F*(3.7, 63.0) = 5.34***, η_p_^2^ = 0.24	*F*(4.2, 71.2) = 4.70**, η_p_^2^ = 0.22	*F*(3.9, 66.5) = 2.89*, η_p_^2^ = 0.15
Target/New CR:			
RT	*F*(1, 17) = 9.11**, η_p_^2^ = 0.35	*F*(1, 17) = 17.53**, η_p_^2^ = 0.51	
VA	*F*(1, 17) = 12.44**, η_p_^2^ = 0.42	*F*(1, 17) = 14.05**, η_p_^2^ = 0.45	
RT × HM		*F*(1, 17) = 7.61*, η_p_^2^ = 0.31	
RT × ST	*F*(2.0, 34.6) = 4.55*, η_p_^2^ = 0.21	*F*(2.2, 37.3) = 10.99***, η_p_^2^ = 0.39	
VA × HM		*F*(1, 17_)_ = 5.69*, η_p_^2^ = 0.25	
VA × ST	*F*(1.3, 21.8) = 5.30*, η_p_^2^ = 0.24	*F*(1.2, 20.5) = 6.37*, η_p_^2^ = 0.27	
RT × AP × HM		*F*(2.0, 33.6) = 9.88***, η_p_^2^ = 0.37	*F*(1.9, 32.9) = 6.44**, η_p_^2^ = 0.27
RT × AP × ST			*F*(3.4, 58.5) = 4.00**, η_p_^2^ = 0.19
VA × AP × ST	*F*(2.8, 47.6) = 4.00*, η_p_^2^ = 0.19	*F*(4.4, 74.7) = 4.81**, η_p_^2^ = 0.22	*F*(4.5, 76.3) = 2.88*, η_p_^2^ = 0.14
VA × AP × HM × ST	*F*(3.7, 62.4) = 5.38**, η_p_^2^ = 0.24	*F*(3.6,61.3) = 3.98**, η_p_^2^ = 0.19	
Nontarget/New CR:			
RT	*F*(1_,_ 17) = 4.51*, η_p_^2^ = 0.21		
VA	*F*(1, 17) = 20.73***, η_p_^2^ = 0.55	*F*(1, 17) = 31.71***, η_p_^2^ = 0.65	*F*(1, 17) = 9.01**, η_p_^2^ = 0.35
RT × HM			*F*(1, 17) = 7.14*, η_p_^2^ = 0.30
RT × ST	*F*(1.2, 21.2) = 4.07*, η_p_^2^ = 0.19	*F*(1, 17) = 8.60**, η_p_^2^ = 0.36	
VA × AP	*F*(1.3, 21.7) = 7.57**, η_p_^2^ = 0.31	*F*(1.2, 20.2) = 4.27*, η_p_^2^ = 0.20	
VA × ST	*F*(1.2, 19.8) = 4.85*, η_p_^2^ = 0.22	*F*(1.2, 19.7) = 6.40*, η_p_^2^ = 0.27	
RT × VA × HM		*F*(1, 17) = 6.66*, η_p_^2^ = 0.28	
RT × AP × HM		*F*(1.5, 25.5) = 7.39**, η_p_^2^ = 0.30	*F*(1.8, 31.0) = 8.24**, η_p_^2^ = 0.33
RT × AP × ST		*F*(4.2, 71.0) = 3.15*, η_p_^2^ = 0.16	*F*(3.6, 61.6) = 5.07**, η_p_^2^ = 0.23
VA × AP × HM		*F*(1.8, 30.5) = 4.29*, η_p_^2^ = 0.20	
VA × AP × ST		*F*(4.1, 69.9) = 5.28**, η_p_^2^ = 0.24	*F*(3.9, 65.8) = 2.75*, η_p_^2^ = 0.14
VA × AP × HM × ST	*F*(3.7, 63.6) = 4.42**, η_p_^2^ = 0.21		
RT × VA × HM × ST	*F*(2.4, 40.0) = 3.50*, η_p_^2^ = 0.17		
Target/Nontarget:			
RT		*F*(1, 17) = 28.27***, η_p_^2^ = .062	*F*(1, 17) = 13.51**, η_p_^2^ = 0.44
VA	*F*(1, 17) = 10.77**, η_p_^2^ = 0.39	*F*(1, 17) = 16.19**, η_p_^2^ = 0.49	
VA × AP	*F*(1.3, 21.6) = 5.84*, η_p_^2^ = 0.26		
VA × ST		*F*(1.2, 20.4) = 6.92**, η_p_^2^ = 0.29	
VA × AP × ST	*F*(3.6, 61.0) = 3.51*, η_p_^2^ = 0.17	*F*(3.9, 66.8) = 4.05**, η_p_^2^ = 0.19	*F*(4.1, 70.5) = 2.91*, η_p_^2^ = 0.15
VA × AP × HM × ST	*F*(3.9, 65.8) = 3.30*, η_p_^2^ = 0.16	*F*(4.0, 68.7) = 3.63**, η_p_^2^ = 0.18	*F*(3.9, 65.5) = 2.75*, η_p_^2^ = 0.14

*p < .5. **p < .01. ***p < .001

### Global analyses

The global analysis between 300 and 500 ms showed a main effect of Response Type, *F*(1.6, 26.8) = 5.49, *p* < .05, η_p_^2^ = 0.24. Pairwise comparison of target hits and new CRs revealed a main effect of Response Type, *F*(1, 17) = 9.11, *p* < .01, η_p_^2^ = 0.35, and an interaction between Response Type × Site, *F*(2.0, 34.6) = 4.55, *p* < .05, η_p_^2^ = 0.21, reflecting greater positivity for targets maximal towards the midline (see Figs. 1 and 2). The effect of Response Type was significant at these sites, *F*(1, 17) = 10.55, *p* < .01, η_p_^2^ = 0.37. A main effect of Response Type, *F*(1, 17) = 4.51, *p* < .05, η_p_^2^ = 0.21, was also obtained in the pairwise comparison of nontarget and new CRs which was moderated by a Response Type × Valence × Hemisphere × Site interaction, *F*(2.4, 40.0) = 3.50, *p* < .05, η_p_^2^ = 0.17, indicating a left-lateralised old/new effect (greater positivity for nontargets) maximal towards the midline. Although the effect at left hemisphere midline sites (F1, C1, P1) was larger in magnitude for negative items, a main effect of Response Type obtained at these sites, *F*(1, 17) = 17.68, *p* < .05, η_p_^2^ = 0.30, was not significantly moderated by Valence. A topographic analysis motivated by the Response Type × Valence × Hemisphere × Site interaction examined whether the nontarget old/new effects elicited by neutral and negative items had different scalp distributions. This was conducted on difference scores obtained by subtracting mean amplitudes of new CRs from nontarget CRs for neutral and negative items, respectively. The data were rescaled using the max-min method to avoid confounding changes in amplitude with changes in the shape of scalp distributions (McCarthy & Wood, 1985). No effects of Valence were observed, indicating that the valence effect observed for nontarget/new CRs in the global contrast was quantitative rather than qualitative. No effects of Response Type were detected in the pairwise comparison of target hits and nontarget CRs.

The global analysis between 500 and 800 ms revealed a main effect of Response Type, *F*(1.4, 24.6) = 10.26, *p* < .01, η_p_^2^ = 0.38, and interactions between Response Type × Anterior/Posterior × Hemisphere, *F*(2.6, 44.0) = 4.51, *p* < .05, η_p_^2^ = 0.21, and Response Type × Anterior/Posterior × Site, *F*(6.1, 104.5) = 2.49, *p* < .05, η_p_^2^ = 0.13. Pairwise comparison of target hits and new CRs showed a main effect of Response Type, *F*(1, 17) = 17.53, *p* < .01, η_p_^2^ = 0.51, and interactions between Response Type × Site, *F*(2.2, 37.3) = 10.99, *p* < .001, η_p_^2^ = 0.39, and Response Type × Anterior/Posterior × Hemisphere, *F*(2.0, 33.6) = 9.88, *p* < .001, η_p_^2^ = 0.37, reflecting greater positivity for targets maximal at left parietal sites towards the midline. A main effect of Response Type was obtained in a confirmatory analysis of data from this maxima (P1), *F*(1, 17) = 20.08, *p* < .001, η_p_^2^ = 0.53. Pairwise comparison of nontargets and new CRs showed interactions between Response Type × Anterior/Posterior × Hemisphere, *F*(1.5, 25.5) = 7.39, *p* < .01, η_p_^2^ = 0.30, Response Type × Anterior/Posterior × Site, *F*(4.2, 71.0) = 3.15, *p* < .05, η_p_^2^ = 0.16, and Response Type × Valence × Hemisphere, *F*(1, 17) = 6.66, *p* < .05, η_p_^2^ = 0.28. These reflected a small positivity for negative and neutral nontargets in the left hemisphere maximal at posterior sites, and greater negativity for neutral nontargets in the right hemisphere (see Figs. 1 and 2). No significant effect of Response Type was obtained at left posterior sites, whereas the post hoc test at right hemisphere sites revealed a Response Type × Valence interaction, *F*(1, 17) = 4.60, *p* < .05, η_p_^2^ = 0.21, reflecting a significant effect of Response Type for neutral items only, Response Type, *F*(1, 17) = 5.38, *p* < .05, η_p_^2^ = 0.24. The pairwise comparison of targets and nontargets revealed a main effect of Response Type, *F*(1, 17) = 28.27, *p* < .001, η_p_^2^ = .062, reflecting greater positivity for targets than for nontargets.

The global analysis between 800 and 1,400 ms showed a main effect of Response Type, *F*(1.6, 27.2) = 4.11, *p* < .05, η_p_^2^ = 0.19, and interactions between Response Type × Anterior/Posterior × Hemisphere, *F*(3.0, 51.0) = 4.17, *p* < .01, η_p_^2^ = 0.20, and Response Type × Anterior/Posterior × Site, *F*(5.4, 91.2) = 3.48, *p* < .01, η_p_^2^ = 0.17. Pairwise comparison of target hits and new CRs revealed crossover interactions between Response Type × Anterior/Posterior × Hemisphere, *F*(1.9, 32.9) = 6.44, *p* < .01, η_p_^2^ = 0.27, and Response Type × Anterior/Posterior × Site, *F*(3.4, 58.5) = 4.00, *p* < .01, η_p_^2^ = 0.19, reflecting greater positivity for targets maximal at right anterior sites and greater negativity for targets at right posterior sites maximal towards the midline. Post hoc tests revealed a main effect of Response Type at right frontal sites, *F*(1, 17) = 4.68, *p* < .05, η_p_^2^ = 0.21, and no effect of Response Type at the right posterior midline site (P2). Pairwise comparison of nontargets and new CRs revealed interactions between Response Type × Anterior/Posterior × Hemisphere, *F*(1.8, 31.0) = 8.24, *p* < .01, η_p_^2^ = 0.33, and Response Type × Anterior/Posterior × Site, *F*(3.6, 61.6) = 5.07, *p* < .01, η_p_^2^ = 0.23, reflecting greater negativity for nontargets maximal at right posterior sites towards the midline. A post hoc test conducted at the right posterior midline site (P2) confirmed that this late posterior negativity was significant, *F*(1, 17) = 9.12, *p* < .01, η_p_^2^ = 0.34. Pairwise comparison of targets and nontargets revealed a main effect of Response Type, *F*(1, 17) = 13.51, *p* < .01, η_p_^2^ = 0.44, reflecting generally greater positivity for targets than for nontargets.

### Planned analyses

ANOVA of ERP data from the four left posterior electrode sites (P7, P5, P3, P1) between 500 and 800 ms incorporated the factors of Response Type (target hits/nontarget CRs/new CRs), Valence (negative/neutral), and Site (inferior/mid-lateral/superior/midline). This analysis revealed a main effect of Response Type, *F*(1.6, 27.7) = 14.53, *p* < .001, η_p_^2^ = 0.46. Subsidiary pairwise comparisons between each pair of Response Types (Bonferonni-corrected *p* values = .017) revealed main effects of Response Type between target hits and new CRs *F*(1, 17) = 25.11, *p* < .001, η_p_^2^ = 0.60 and between target hits and nontarget CRs *F*(1, 17) = 16.82, *p* = .001, η_p_^2^ = 0.50, but not between nontarget CRs and new CRs (see Fig. 3). No interactions were observed between Valence and Response Type.

The planned analysis of ERP data from the four right frontal electrode sites (F8, F6, F4, F2) between 800 and 1,400 ms incorporated the factors of Response Type (target hits/nontarget CRs/new CRs), Valence (negative/neutral), and Site (inferior/midlateral/superior/midline). This analysis revealed a main effect of Response Type, *F*(1.9, 32.1) = 3.66, *p* < .05, η_p_^2^ = 0.18, and a Response Type × Valence interaction, *F*(1.9, 33.1) = 4.27, *p* < .05, η_p_^2^ = 0.20. The following six post hoc tests (Bonferonni-corrected *p* values = .008) were conducted: (i) targets/new CRs, (ii) nontargets/new CRs, (iii) targets/nontargets (all incorporating factors of Response Type, Valence, and Site), and (iv) negative/neutral targets, (v) negative/neutral nontargets, (vi) negative/neutral new CRs (all incorporating factors of Valence and Site). The only significant right frontal effects obtained in these analyses were a main effect of Valence in the negative/neutral nontargets contrast, *F*(1, 17) = 9.03, *p* = .0079, η_p_^2^ = 0.35 (greater positivity for negative nontargets; see Fig. 4) and a marginal Response Type × Valence interaction, *F*(1, 17) = 8.65, *p* = .009, η_p_^2^ = 0.34, in the contrast between target hits and nontarget CRs, reflecting a significant target/nontarget effect for neutral items only, *F*(1, 17) = 16.64, *p* < .001, η_p_^2^ = 0.49 (see Figs. 3 and 4).

## Discussion

### Behaviour

This experiment employed the paradigm used in the high target accuracy condition from (Herron & Rugg, 2003b) to examine whether the recollection of negatively valenced words could be controlled to the same degree as neutral stimuli. Although all task parameters were maintained from this earlier study, average target accuracy was slightly higher here (.82) than previously (.76). One explanation for this increase is that the nature of the encoding task performed on targets in Study 2 (pleasantness rating) arguably became more salient here with the introduction of overtly negative words. Discrimination of targets from new items was higher for neutral than for negative stimuli, and response bias was more liberal for negative than for neutral stimuli, largely because participants were more likely to false alarm (i.e. incorrectly make a ‘target’ response) to negative items than to neutral items. This was the case for both new items and for nontargets. This partially replicates previous findings that emotionally valenced items are associated with a more liberal response bias than neutral items (Dougal & Rotello, 2007; Thapar & Rouder, 2009; Windmann & Kutas, 2001), although the behavioural effects of valence were not global here because negative targets did not attract more correct responses than neutral targets. Similarly, whereas reaction times associated with target responses were statistically equivalent, those associated with nontarget CRs and new CRs were significantly longer for negative than for neutral words, indicating that information diagnostic of a correct response took longer to accrue for negative items.

Interestingly, there was no behavioural evidence for the superior recollection of negative items. Indeed, if it is assumed that nontargets are correctly classified on the basis of recollection (although the ERP data do not support this assumption, as will be discussed below), the behavioural data would point towards the superior recollection of source information for neutral words given the higher levels of accuracy for neutral than for negative nontargets. At first sight this seems surprising given previous reports of superior source judgments for emotionally arousing stimuli when participants were asked to recall the colour or location of stimuli during encoding (D’Argembeau & Van der Linden, 2004; Doerksen & Shimamura, 2001; Kensinger & Corkin, 2003). However, it has been demonstrated that this source memory superiority does not extend to emotionally valenced items when source judgments based on encoding task are required (Kensinger & Schacter, 2006), as is the case in the present study. Kensinger and Schacter (2006) also reported that emotionally arousing items were more likely to be recognised but attributed to the wrong encoding task, which was also the case here for nontargets. It has been argued on the basis of these disparate findings that while emotional arousal enhances source judgments for intrinsic within-object characteristics such as colour or location, this does not extend to extrinsic characteristics such as encoding task (Mather, 2007). The present findings are consistent with this hypothesis.

The behavioural data are consistent both with the suggestion that negative words are more susceptible to false memory due to enhanced semantic cohesiveness amongst items (Maratos et al., 2000; White et al., 2014) and with the finding that emotionally valenced items are associate with deficits in source monitoring processes (Cook et al., 2007). The observed effect of valence on response bias is surprising given that negative and neutral items were intermixed within the same test block, and it appears that this was predominantly driven by selective effects of valence on memory accuracy (and associated RTs) for nontargets and new items, while target accuracy and RTs were left unaffected by valence. The false memory hypothesis above does not therefore provide a full explanation for the behavioural findings given the absence of this effect for targets. It is likely that the valence-oriented encoding task performed for targets encouraged participants to focus upon retrieving their subjective valence judgments assigned at study. This interpretation is consistent with the idea that exclusion memory tasks encourage participants to adopt target-centric ‘retrieval orientations’ (Rugg & Wilding, 2000), which influence the processing of test items so as to facilitate the accurate retrieval of information from the target encoding phase, and that these processes also lead to the reduced recollection of nontargets (Herron & Rugg, 2003b). In the present study, attempting to recapitulate (or recollect) the valence judgments previously made for each item may have resulted in more accurate recollection of item-specific information for those items actually studied in the valence task (i.e. targets), rendering them less susceptible to the valence effects observed for nontargets and new items.

### ERPs

A robust left parietal old/new effect was observed for targets between 500 and 800 ms, replicating Herron and Rugg (2003b). The finding that correctly classified targets in the exclusion task consistently elicit significant left parietal old/new effects (Czernochowski et al., 2005; Dzulkifli & Wilding, 2005; Dzulkiflil et al., 2006; Evans et al., 2010; Herron & Rugg, 2003a, 2003b; Herron & Wilding, 2005; Rosburg et al., 2013; Rosburg et al., 2011) adds to the large body of research linking this effect with recollection, because correct target responses are predominantly made as a result of recollecting source specifying information from the target encoding phase. The literature regarding the influence of emotional valence on the left parietal old/new effect is mixed, and our finding that the target left parietal effect was unaffected by valence replicates Windmann and Kutas (2001). Conflicting evidence that left parietal old/new effects for negatively valenced words were either smaller (Maratos et al., 2000) or larger (Inaba et al., 2005) than those elicited by neutral words were driven by differential effects of valence on studied and unstudied items, whereas valence did not differentially modulate ERPs for each response type between 500 and 800 ms in this study (valence instead exerted a global effect as can be seen in Table 2). It should also be acknowledged that the three prior ERP studies examining the influence of word valence on the left parietal old/new effect employed simple recognition judgments rather than judgments based on source, and that recollection may therefore have made smaller and more variable contributions to memory responses across these studies than here. The invariance of the left parietal old/new effect across valence converges with A. P. Smith et al.’s (2004) findings using objects and the source memory experiment using neutral words encoded in emotional sentence reported by Maratos and Rugg (2001). This finding is again consistent with the hypothesis that recollection of contextual information external to the stimulus is not enhanced by stimulus valence (Kensinger & Schacter, 2006; Mather 2007).

Critically, whereas both classes of targets elicited large and reliable left parietal old/new effects, these were largely absent for both neutral and negative nontargets. It is important to note here that although the false alarm rate was higher for negative nontargets, nontarget ERPs were conditionalised purely upon correct responses. Because the same response key is employed for both nontargets and new items, the question is whether correct nontarget responses are based upon recollection (‘recall-to-reject’; Clark, 1992; Rotello & Heit, 2000) or upon an absence of recollection. Despite the deeply elaborative encoding of nontargets, the ERP findings indicate that recollection levels associated with correct nontarget responses were significantly and equivalently reduced for both negative and neutral nontargets (relative to correct target responses), and to such an extent that significant correlates of recollection were not evident at all (or ‘gated’; Morcom & Rugg, 2012) for either neutral or negative nontargets.

Significant left parietal effects were also evident between targets and nontargets for both neutral and negative items, and the magnitude of this effect was not influenced by emotional valence. This replicates the findings of Herron and Rugg (2003b, Experiment 1) while extending them to negatively valenced words. When accounting for these original findings, Herron and Rugg (2003b) conducted an additional behavioural study in which Study 1 items (previously nontargets) were designated as targets to verify the memorability of these items. A target hit rate of .86 was reported alongside a new item false alarm rate of 0.03, confirming that source memory for these items was very high (Herron & Rugg, 2003b). Given the high levels of memorability associated with these items (and the deeply elaborative nature of the sentence generation encoding task), the attenuation of the left parietal effect associated with these items is highly unlikely to be due to forgetting. The presence of significant old/new effects for both targets and nontargets in the earlier 300–500-ms epoch supports this assertion, as does the RT data showing significantly longer RTs for nontarget than new CRs, indicating that all studied items were initially recognised. Furthermore, the late posterior negativity (LPN) was observed for both classes of nontargets relative to new CRs. This effect is thought to comprise a combination of action monitoring processes engaged when a recognised item requires a negative response and processes involved in the search for source specifying information (Herron, 2007; Johansson & Mecklinger, 2003; Mecklinger et al., 2016). The presence of the LPN for nontargets, therefore, indicates that these items were consciously recognised to some degree. The account previously given for this pattern of data is that the requirements of the exclusion task (to identify targets on one button and respond to both nontargets and new items on the alternate button) induces a retrieval strategy in which participants focus exclusively on the presence or absence of diagnostic source information from the target encoding phase for all recognised items, that participants are more likely to adopt this strategy when target memorability is high and reliable, and that such a strategy reduces the likelihood of nontarget recollection (Herron & Rugg, 2003b).

Rosburg and colleagues (2011; Rosburg et al., 2013) proposed an interesting modification to this account by suggesting that nontargets in exclusion tasks are more likely to give rise to left parietal old/new effects as their ‘ease of accessibility’ increases, and that nontarget retrieval ‘might actually be driven primarily by bottom-up mechanisms, in the sense that subjects do not actively search for source information of nontargets, but that the presentation of nontarget cues reactivates this information’ (Rosburg et al., 2011, pp. 2966). Their proposal was motivated by the finding that the amplitude of the nontarget left parietal old/new effect was positively correlated with the memorability of items from both sources in an individual differences analysis, and supported by the further finding that items from the same source elicited left parietal effects that were positively correlated when they were designated as targets and nontargets in different conditions (Rosburg et al., 2013). The authors argued that, in some cases, nontarget retrieval may occur even when these items are not strategically emphasised because they are simply easier to remember than targets and are incidentally recollected (Rosburg et al., 2011). If this account is correct, the present findings indicate that neither negative emotional valence nor the higher arousal values associated with these items are sufficient to drive these bottom-up mechanisms and reactivate incidental recollection. This is despite the fact that the behavioural experiment reported by Herron and Rugg (2003b) indicated that memorability of items from Study Phase 2 should be slightly higher (.86 uncorrected, Pr = .083) than the memorability of targets.

The present findings also speak to, and extend, those obtained from the directed forgetting literature. In these paradigms, participants encode pairs of items and are then instructed to either ‘think’ (i.e. remember) or ‘don’t think’ of the paired associate of each item presented at test, with the repeated suppression of ‘don’t think’ items reducing their memorability during subsequent recall (Anderson & Green, 2001). A series of ERP studies have shown that the amplitude of the left parietal old/new effect associated with recollection is reduced for ‘don’t think’ relative to ‘think’ items when participants are instructed to suppress these memories, providing converging evidence that recollection is subject to executive control (Bergstrom et al., 2009a, 2009b; Bergstrom et al., 2007; Depue et al., 2013; Hanslmayr et al., 2009; Mecklinger et al., 2009). Although it is not yet known whether the left parietal old/new effect can be suppressed for emotionally valenced items in directed forgetting paradigms, van Schie, Geraerts, and Anderson (2013) reported behavioural evidence that the recollection of neutral and negatively valenced items were equally suppressible during directed forgetting, and that self-reports indicated that the majority of participants controlled recollection via direct suppression (i.e. letting their mind go blank or repeating the presented word). The present findings extend those of van Schie et al. (2013) in that there was no explicit requirement to suppress or inhibit nontarget memories, and yet neural correlates of recollection were significantly attenuated for negative words when these were designated as nontargets. Theories of strategic retrieval instead emphasise the role of proactive memory control processes in which the contents of the target encoding task are prioritised over the nontarget encoding task, forming part of a target-specific retrieval orientation and thereby reducing nontarget retrieval. The present findings provide novel evidence that the left parietal old/new effect can be suppressed for emotionally valenced items, and that explicit directed forgetting instructions are not required to obtain this finding.

A second aim of the present study was to examine ERP correlates of postretrieval monitoring to assess the impact of emotional valence on this stage of memory processing. Although the effect between targets and new CRs did not survive the conservative correction applied during the planned analyses at right frontal sites, the post hoc analysis conducted at right frontal sites in the global analyses indicated that targets were more positive going than new CRs and that this effect was not influenced by valence. Evidence of right frontal effects for correctly classified targets is uncontroversial and consistent with a source monitoring account of the effect. Of greater interest was the finding that a large and statistically robust right frontal effect was elicited by neutral targets when compared with neutral nontargets, but that this effect was not evident between negative targets and nontargets (see Fig. 4). Rather than reflecting a source monitoring deficit for negative items, however, negative targets and nontargets did not diverge at right frontal sites because negative nontargets also elicited a focal and robust right frontal positivity when compared with neutral nontargets (see scalp map in Fig. 4). To clarify, the results suggest that neutral targets, negative targets, and negative nontargets all elicited right frontal effects whereas neutral nontargets did not.

The finding that negative nontargets required a greater degree of postretrieval monitoring than their neutral equivalents is consistent with the behavioural results showing that the correct rejection rate was lower for negative than for neutral nontargets and that it took longer to correctly identify negative than neutral nontargets. It is also notable that no right frontal effect was evident between negative and neutral targets, and that the behavioural data converges with this observation with neither target hit rates nor response times differing between negative and neutral targets. The absence of a right frontal effect between neutral nontargets and new CRs is consistent with the view that participants failed to retrieve (i.e. ‘gated’) or evaluate nontarget source information for neutral items, whereas the behavioural and ERP data suggest that negative nontargets engaged source monitoring processes even in the absence of robust ERP indices of recollection. Whether the enhanced monitoring evident for negative nontargets was due to negative valence per se or to the greater semantic relatedness of negative words (Maratos et al., 2000; White et al., 2014), this effect of valence was not observed for new item correct rejections. This indicates that participants only engaged postretrieval monitoring for negative items identified on some level as being old, arguably on the basis of familiarity-based recognition. It is possible that postretrieval monitoring extended beyond the end of the 1,400-ms recording epoch employed here, particularly for negative targets and nontargets which were associated with average RTs that extended beyond 1,400 ms. However, the fact that a significant right frontal effect was observed for negative nontargets (which were associated with the longest RTs at 1,733 ms) relative to neutral nontargets indicates that at least the early portion of this effect should have been captured if it was present between response types.

Supporting the view that nontargets were recognised on the basis of familiarity, it was found that nontarget old/new effects were significant in the 300–500-ms epoch. ERP memory effects within this time window are widely considered to reflect familiarity-based recognition (Rugg & Curran 2007; Wilding & Ranganath, 2011, but see Voss & Paller, 2006, for a conceptual priming account), and this finding therefore suggests that nontargets were recognised on the basis of familiarity before recollection was attenuated. There was some evidence that these earlier familiarity processes were engaged to a greater extent for negative than for neutral nontargets (as indicated by a higher order interaction in the global analyses), whereas valence did not influence the target old/new effect between 300 and 500 ms. It is possible that the strategic control of nontarget memories began during this epoch, attenuating the neutral nontarget familiarity effect while this effect was more resistant to strategic control for negative items. Target old/new effects, of course, would not have shown an attenuation for either neutral or negative items. This interpretation, however, is limited both by the finding that valence did not significantly influence the nontarget old/new effect in the post hoc or topographic analyses, and by the absence of significant differences between targets and nontargets in this time window.

One constraint of the present study was that the list lengths required to obtain desirable levels of memory performance while maintaining an adequate signal-to-noise ratio precluded the inclusion of overtly positively valenced stimuli, and it therefore remains unknown whether the strategic control of emotional memory extends to this class of stimuli. On a related note, there were insufficient trials to perform a four-way separation of nontarget ERPs according to the precise valence rating assigned by participants during study. Negative nontargets were instead classified on the basis of ANEW ratings in the present study, and while 97% of these items attracted subjective ‘fairly unpleasant’ or ‘very unpleasant’ ratings, it would be interesting to investigate whether the present findings extend to nontargets attracting extremely negative subjective valence ratings and whether there is a graded effect according to subjective levels of valence. Finally, it would also be of interest to determine whether neutral nontargets embedded in a negative context at encoding (e.g. Maratos & Rugg, 2001) would elicit ERP indices of incidental recollection.

In conclusion, it was found that healthy young adults were able to prioritise their recollection of targets over nontargets for both neutral and negatively valenced words. This was demonstrated by left parietal old/new effects for both neutral and negative targets relative to nontargets and new words. Participants achieved this so effectively that no significant left parietal old/new effects were detected for either neutral or negative nontargets, despite the fact that these items were encoded in a deeply elaborative task and equivalent items had elicited very high levels of recollection when they served as targets in a previous behavioural study. This extends the strategic retrieval literature by showing that memories associated with negatively valenced test stimuli are subject to strategic control, and that these items do not automatically reactivate recollection via a bottom-up mechanism despite the higher arousal values associated with these items. These findings also extend the directed forgetting literature by demonstrating that the recollection of negatively valenced words can be attenuated without recourse to explicit direct suppression or thought substitution instructions. The finding that behavioural and ERP measures of target recollection were unaffected by valence supports the view that recollection of extrinsic contextual information is not moderated by stimulus valence. Finally, although target right frontal old/new effects were unaffected by valence, these were detected between targets and nontargets for neutral items only. The finding that negative nontargets also elicited enhanced positivity at right frontal sites relative to neutral nontargets indicates that negative nontargets required a greater degree of source monitoring than their neutral counterparts.

## Footnotes

1 Although only neutral and negative stimuli were drawn from the ANEW database, this four-way pleasantness judgment response schedule was retained from the design employed by Herron and Rugg (2003b) despite the absence of overtly positive stimuli. Our earlier study did not employ a fifth ‘neutral’ response as it was observed during piloting that forced pleasant/unpleasant judgments resulted in higher levels of subsequent memory.
